# Vitamin blood concentration and vitamin supplementation in bottlenose dolphins (*Tursiops truncatus*) in European facilities

**DOI:** 10.1186/s12917-016-0818-1

**Published:** 2016-09-05

**Authors:** Angela Emilia Ricarda Gimmel, Katrin Baumgartner, Annette Liesegang

**Affiliations:** 1 Institute of Animal Nutrition, Vetsuisse Faculty, University of Zurich, Winterthurerstrasse 270, 8057 Zurich, Switzerland; 2 Zoo Nuremberg, Am Tiergarten 30, 90480 Nuremberg, Germany

**Keywords:** Vitamin supplementation, Bottlenose dolphin, *Tursiops truncatus*, Vitamin blood concentration, Fish thawing, Retinol, Thiamine, Calcidiol, Tocopherol, Cobalamin

## Abstract

**Background:**

As fish eaters bottlenose dolphins (*Tursiops truncatus*) in human care need to receive daily vitamin supplementation, because whole thawed fish lacks certain vitamins. However, the exact concentration of supplementation has not been established and is a matter of discussion. To ensure adequate vitamin supplementation in pets, vitamin blood concentrations are measured. This is not a common practice in dolphins. The objective of the present study was to collect information about vitamin supplementation in bottlenose dolphins and on vitamin blood concentrations of healthy animals in European facilities. In addition, these results were compared with blood levels of wild animals. Conclusions on how to provide bottlenose dolphins in human care with an effective vitamin supplementation will then be drawn.

Initially, fish-handling techniques and vitamin supplementation were evaluated by questionnaire, which was sent to 25 European facilities that house bottlenose dolphins. Secondly, blood samples from 57 dolphins living in 10 facilities were taken and sent by mail to a reference laboratory. They were analysed for retinol, thiamine pyrophosphate, cobalamin, calcidiol and tocopherol. The blood concentrations were then correlated with vitamin supplementation, fish handling techniques and pre-existing blood concentrations of free-ranging dolphins. Finally, the data was subjected to a standard analysis of variance techniques (ANOVA) and a linear model analysis.

**Results:**

Fish was mainly thawed in a refrigerator. Further, the 95 % confidence interval for retinol blood concentrations was 0.048 to 0.059 mg/l and for tocopherol 17.95 to 20.76 mg/l. These concentrations were 27 and 53 %, respectively, higher than those found in free-ranging animals. In contrast, calcidiol concentrations (143.9–174.7 ng/ml) of the dolphins in human care were lower than in blood found for free-ranging animals. Regarding thiamine pyrophosphate and cobalamin, concentrations ranged between 0.42 and 0.55 mg/l and 175.55 and 275.22 pg/ml respectively. No reference concentrations for free-ranging *Tursiops truncatus* were found.

**Conclusions:**

These findings suggest an over-supplementation of retinol (vitamin A) and tocopherol (vitamin E) in bottlenose dolphins (*Tursiops truncatus*) housed in human care. Therefore, vitamin A supplementation should not exceed 50,000 IU per animal per day and vitamin E supplementation should be around 100 IU per kg fed fish per day.

**Electronic supplementary material:**

The online version of this article (doi:10.1186/s12917-016-0818-1) contains supplementary material, which is available to authorized users.

## Background

In 2015, 255 bottlenose dolphins (*Tursiops truncatus truncatus*) were housed in 26 European facilities (Hartmann, personal communication, 2015). These dolphins are kept for scientific research, human-dolphin therapy, entertainment, as well as educational and conservational purposes. As knowledge about cetacean husbandry has increased, so has the necessity to provide the highest possible standards of animal husbandry to ensure health, reproductive success and general animal welfare. Nutrition is a fundamental component of animal welfare since malnutrition impairs the biological functioning of organisms; it arises when animals are fed inadequate or inadequately balanced food [1]. Bottlenose dolphins in human care receive whole thawed fish as feed. The term “fish” as used here includes all fish that may be fed to fish-eating animals, including saltwater fish and other seafood items (squid, clams, etc.) [2]. Whole fresh fish represents a complete and balanced diet for cetaceans and covers their nutritional requirements in the wild. In terms of vitamins, it naturally contains an abundance of fat soluble vitamins, like vitamin A (retinol), vitamin D3 (calcidiol) and vitamin E (tocopherol) [3]. While free-ranging dolphins are known to eat a variety of different species of fish (including molluscs and crustaceans [4, 5]), which change seasonally and regionally, this variety is often impaired in human care. These dolphins receive a smaller selection of fish species that were caught, frozen, stored and then thawed before feeding. Hence, during this process (mainly during storing and thawing) nutrient concentration decreases [2]. The extent of these nutrient losses and the question how they are affected by thawing have not been thoroughly investigated to date. Since fish is potentially hazardous feedstuff and spoils quickly if not handled properly, special care in food-handling procedures is crucial to its nutritive quality.

Vitamin E, tocopherol, is a fat-soluble vitamin that acts as an antioxidant. The vitamin E concentration of whole fish depletes during fat peroxidation, the result of which is known as rancidity. When fat which is present in a high proportion in many fish species (mainly as long-chain polyunsaturated fatty acids) reacts with oxygen, free fatty radicals are produced. Once produced, they have the ability to react with other fatty acids, resulting in a chain reaction. Vitamin E acts as an antioxidant to stop the cycle, protecting the cells from oxidative injury. The more long-chain polyunsaturated fatty acids a fish contains, the faster is the decline in vitamin E, and other antioxidants like vitamin C (ascorbic acid) and selenium. The same principle applies to storage time. The longer a fish is stored, the more vitamin E gets depleted. Vitamin E deficiency in animals leads to steatitis, muscular degeneration, liver necrosis and anaemia [3]. It is thus very important to supplement frozen and thawed fish with vitamin E. The recommendations for vitamin E supplementation are 100 IU per kg fed fish per day for marine mammals [3].

Most species of fish contain an abundance of the other fat soluble vitamins, such as vitamin A and vitamin D3 [2]. Extra supplementation of both vitamins may not be advisable. Especially fat soluble vitamins get stored in different fatty deposits and can thus accumulate in the body. A study in fur seals suggests, that excess vitamin A supplementation inhibits the uptake of vitamin E [6].

Vitamin B1, thiamine pyrophosphate, is broken down by the enzyme thiaminase, which is naturally present in many fish species, such as herring, smelt and mackerel. Due to the fact, that this enzyme activity slows down, but still continues to metabolize while being frozen, more vitamin B1 is broken down when a fish is stored for a longer time. It is well established, that frozen and thawed fish has to be supplemented with thiamine before feeding, as deficiencies result in neurological symptoms such as tremors, head shaking or star gazing and can lead to death [3]. The recommendations for vitamin B1 supplementation are 25 mg per kg fed fish per day for marine mammals [3].

Another point of interest for water soluble vitamins is related to the losses that occur during fish thawing. During the defrosting of fish water is always lost. Water soluble vitamins are thus, by consequence, often reduced in the thawing process through washing out, especially when fish is thawed using running water or when fish is rinsed with water after thawing [2]. The vitamins are also flushed out with fluids seeping from surfaces when the fish is cut into small pieces for feeding.

To summarise, it is essential for the health of the bottlenose dolphins to provide vitamin supplementation when the diet consists only of whole thawed fish. The exact knowledge on which vitamins should be supplemented and in which concentrations has not been fully established. In many facilities that keep marine mammals, this is a matter of intense discussion. Many institutions rely on multi-vitamin tablets provided by different companies. These often supply a wide range of vitamins and minerals. Although under-supplementation and clinical problems as described above are rarely observed, over-supplementation (especially of fat soluble vitamins) might be an issue with multi-vitamins. To ensure adequate vitamin supplementation in pets, blood concentrations are often measured. Few vitamin blood level-baselines for bottlenose dolphins have been established to date in wild animals, two of them being serum retinol and α- and γ-tocopherol [7]. The objective of the present study was to gather information on vitamin supplementation of bottlenose dolphins and on vitamin blood concentrations of healthy animals in European facilities. In addition, these obtained values were compared with blood levels of wild animals. Vitamin blood concentrations in European facilities were tested against different individual parameters such as age, sex, pregnancy status, lactation status, indoor/outdoor housing, vitamin supplementation and thawing method of fish. From the evaluated levels, recommendations on how to provide effective vitamin supplementation in bottlenose dolphins were formulated.

## Methods

A questionnaire was designed and distributed to 25 European facilities which keep bottlenose dolphins (*Tursiops truncatus truncatus*). All institutions are members of the European Association of Aquatic Mammals (EAAM). The questionnaire was divided into four different sections, i.e. ordering and purchasing, storing and thawing fish, handling thawed fish, and feeding. In the last section of the questionnaire, the daily vitamin supplementation for each individual dolphin was collected as well as individual parameters such as age, sex, whether the dolphin pools are indoor or outdoor, and whether the females are lactating or pregnant (see Additional file 1: Questionnaire). A total of 19 questionnaires were returned and ten facilities agreed to provide voluntary blood samples of their animals to be tested for vitamin blood concentrations. A total number of 57 bottlenose dolphins were sampled on a voluntary basis during morning medical training sessions: 33 females (five pregnant, eight lactating) and 24 males. A total number of nine juveniles and 48 adults, and of 17 indoors and 40 outdoor animals were tested.

The blood samples were obtained from March 2007 to December 2012 and each animal was sampled once. Blood was drawn from the tail fluke by veterinary staff in the morning medical training sessions. The staff were instructed to draw blood before the morning feeding, so that all animals were fasted overnight prior to blood sampling. All institutions approved of this type of sampling. Two millilitres of serum and 1 ml of EDTA blood were collected, kept cool and in the dark, and shipped to the reference laboratory within 2 days (Vet Med Labor GmbH, Division of IDEXX Laboratories, Ludwigsburg, Germany). The reference laboratory determined blood concentrations of retinol, thiamine pyrophosphate, cobalamin, calcidiol and tocopherol. HPLC/U-HPLC analyses have been done on a U-HPLC system from Dionex-RS LC 3000 with a diode array detector (for retinol: Detection wavelength 325 nm, calcidiol: Detection wavelength 265 nm and tocopherol: Detection wavelength 295 nm) and a fluorescence-detector (for thiamine pyrophosphate detection: Extinction 367 nm/Emission 435 nm and cobalamin detection: Extinction 320 nm/Emission 415 nm). The sample preparation for the HPLC run for retinol, calcidiol, tocopherol, thiamine pyrophosphate and cobalamin was done according to the standardized method from Chromsystems. Cobalamin was first determined with ECLIA (Electro-chemiluminescence immunoassay), but the testing device in the laboratory was changed in October 2012, so the last 5 samples of cobalamin were analysed with CLIA (Chemiluminescent Immunoassay). Detection limits were the following: Retinol 0.02–2.25 mg/l, thiamine pyrophosphate 0.028–900 mcg/l, cobalamin ECLIA 30–2000 pg/ml or CLIA 45–2000 pg/ml, calcidiol 1.4–250 mcg/l and tocopherol 5–45 mg/l.

The data were analysed using standard analyses of variance techniques (ANOVA), including statistical validity checks on the Normality of the residuals. It was verified that the different experimental groups had similar variance (homogeneity of variance checks). Each of the serum vitamin concentrations (retinol, thiamine pyrophosphate, cobalamin, calcidiol and tocopherol) were measured against the following parameters: age, sex, pregnancy status, lactation status, indoor/outdoor housing, vitamin supplementation and thawing method. If either of these checks failed, the data was transformed by taking logarithms and the analyses and validity checks were repeated. When a significant result occurred, that data was checked with a linear model, where the institution was included as a fixed effect.

## Results

### Questionnaire

Out of 25 questionnaires 19 were returned. The results are presented in Table 1. It becomes evident that over 60 % of the institutions feed five or more fish species. One institution varied their fish to match seasonal availability. The species of fish that are fed the most are herring, capelin and squid. Fourteen institutions checked the history of the fish, i.e. for example the place of origin or the time when it was caught. More than half of the participating institutions used the same company for their fish supplies. All facilities ordered fish block frozen: rectangular frozen blocks that contain one single species of fish. Three facilities additionally ordered individually frozen fish. All institutions conducted nutritional analyses before feeding the fish. These mainly included caloric and fat content, protein, crude ashes, peroxide and microbial content. So far, no whole fish vitamin analysis has been done by the participating facilities. Thawing techniques varied greatly, with thawing in a refrigerator used most. The different thawing techniques are summarized in Table 2.

**Table 1:** Results of the questionnaire issued to 19 European facilities that keep bottlenose dolphins (*Tursiops truncatus*)

Questionnaire section			Number	[%]
Ordering and purchasing	Number of fish species ordered	2–4	7	37
		5–6	11	58
		>6	1	5
	Fish species fed	Herring	16	^a^
		Squid	16	
		Capelin	16	
		Sprat	13	
		Mackerel	11	
	Fish history checked	Yes	14	74
		No	5	26
	Fish wholesaler used	Wholesaler A	10	53
		Wholesaler B	4	21
	Fish freezing method	Block frozen	19	
		Individually frozen	3	
	Fish block size	1–10 kg	7	
		10.1–20 kg	14	
		20.1–40 kg	11	
	Shipment inspection	Documentation	13	
		Sings of thawing	14	
		Temperature	10	
		Manually for firmness	11	
Storing and thawing	Nutritional analysis conducted	With every shipment	15	79
		Every 3 months	4	21
	Vitamin analysis conducted		0	0
	Storage time	1–2 months	4	21
		3–6 months	10	53
		7–12 months	5	26
Handling thawed fish	Time before feeding	<24 h	18	95
		24–48 h	1	5
Feeding	Fish preparation	Cutting	6	32
		No cutting	13	68

*N* Number of institutions, ^a^if more than one answer was given, no percentage could be calculated

**Table 2:** Different thawing techniques used in European facilities to thaw fish for bottlenose dolphins (*Tursiops truncatus*)

Number	Thawing technique used	Time (if specified)
6	Thawing in a refrigerator only	Overnight (12–14 h)
4	Thawing first in refrigerator, then under running water	
3	Thawing at room temperature only	Overnight (12–14 h)
3	Thawing first at room temperature, then under running water	
2	Thawing first in refrigerator, then at room temperature	
1	Thawing under running water only	

*N* Number of institutions using this thawing techniques

After thawing, six facilities cut their fish into smaller pieces before feeding. Five institutions have tested vitamin blood concentrations in bottlenose dolphins so far, but none on a regular basis. All of the facilities draw blood during medical training sessions at least twice a year, if not more often.

**Fig. 1:**
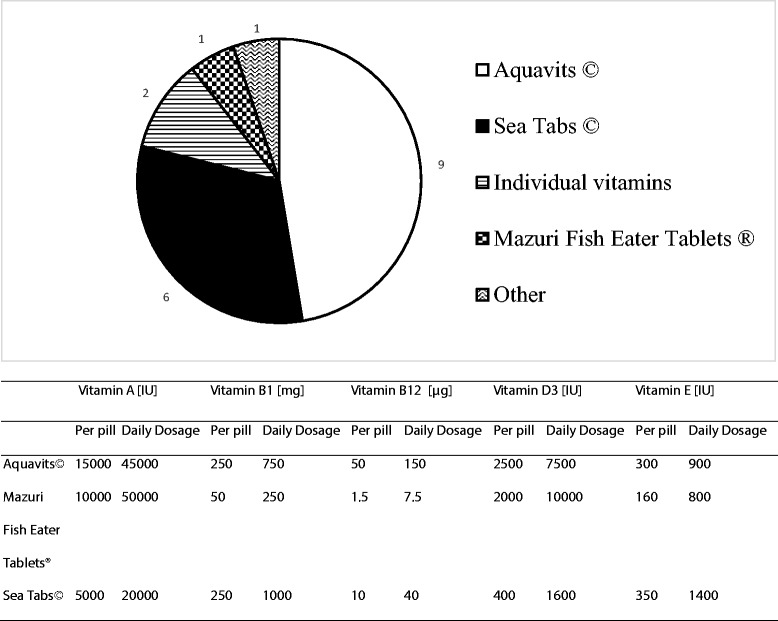
Brand names, composition and daily supplier’s recommendation of vitamin supplements used for bottlenose dolphins (*Tursiops truncatus*) in 19 European facilities. Aquavits © produced by Zoovet Products, IZVG LLP, West Yorkshire, United Kingdom. SeaTabs © produced by Pacific Research Laboratories, Inc., California, United States. Mazuri Fish Eater Tablets ® produced by Mazuri Zoo Foods, Essex, United Kingdom

Different vitamin brands are used for supplementation in different institutions. Their distribution as well as the vitamin content of different pills and daily vitamin recommendations are outlined in Fig. 1.

A total of 139 animals were in the care of the 19 facilities that have answered the questionnaire.

### Blood analysis

The 95 % confidence interval of the 57 bottlenose dolphins in human care was higher in retinol and tocopherol than in samples of free-ranging animals (see Table 3). The animals in human care showed 27 and 53 % higher values regarding these two vitamins. By contrast, the concentration of calcidiol was 27 % lower than the reference levels found in the literature.

**Table 3:** 95% confidence interval of vitamin blood concentrations of bottlenose dolphins (*Tursiops truncatus*)

Status	Number	Retinol [mg/l]	Thiamine pyrophosphate [mg/l]	Cobalamin [pg/ml]	Calcidiol [ng/ml]	Tocopherol [mg/l]
In human care	57	0.048–0.059	0.42–0.55	175.55–275.22	143.9–174.7	17.95–20.76
Reference free-ranging^a^	55	0.036–0.046				12.56–12.66
Reference free-ranging^b^	2				293.1	
Reference in human care^c^	3				197.1–222.3	

*N* Number of individual bottlenose dolphins (*Tursiops truncatus*) tested

^a^Values from Crissey, Wells 1999, ^b^Values from Keiver, Ronald, 1988, ^c^Values from Slifka, Crissey, Nutrition Advisory group 2001

**Fig. 2:**
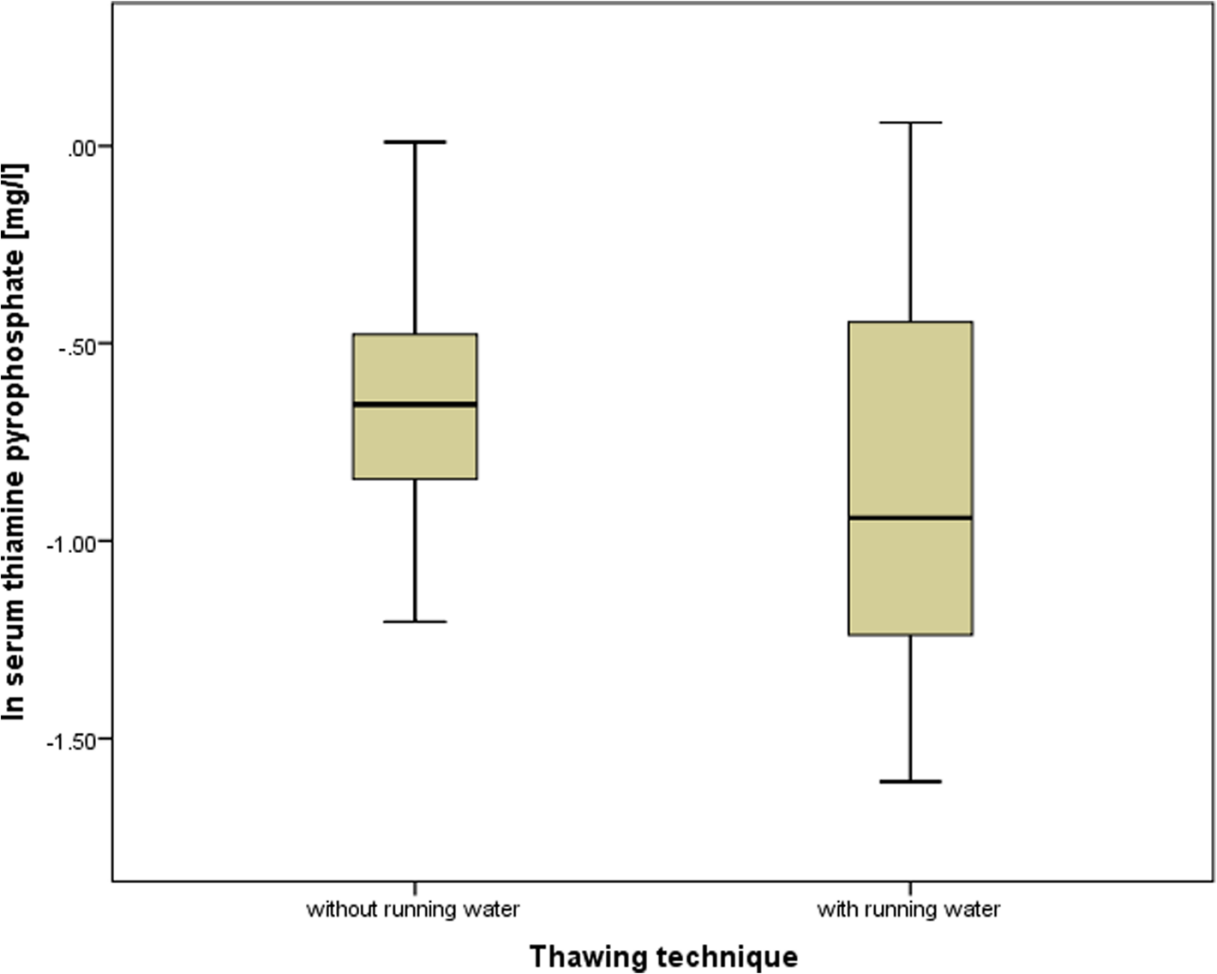
Thiamine pyrophosphate serum concentrations of bottlenose dolphins (*Tursiops truncatus*) in relation to thawing techniques. *Box* plots comparing thawing techniques in relation to the natural logarithm of thiamine pyrophosphate serum concentrations of bottlenose dolphins (*Tursiops truncatus*). *N* = 25 for thawing without running water (thawing in refrigerator, at room temperature), *N* = 29 for thawing with running water (includes any thawing method that uses running water). *Box* plots show the range of the mean 50 % of the data. Within the *boxes*, the *thick line* indicates the arithmetic mean

**Fig. 3:**
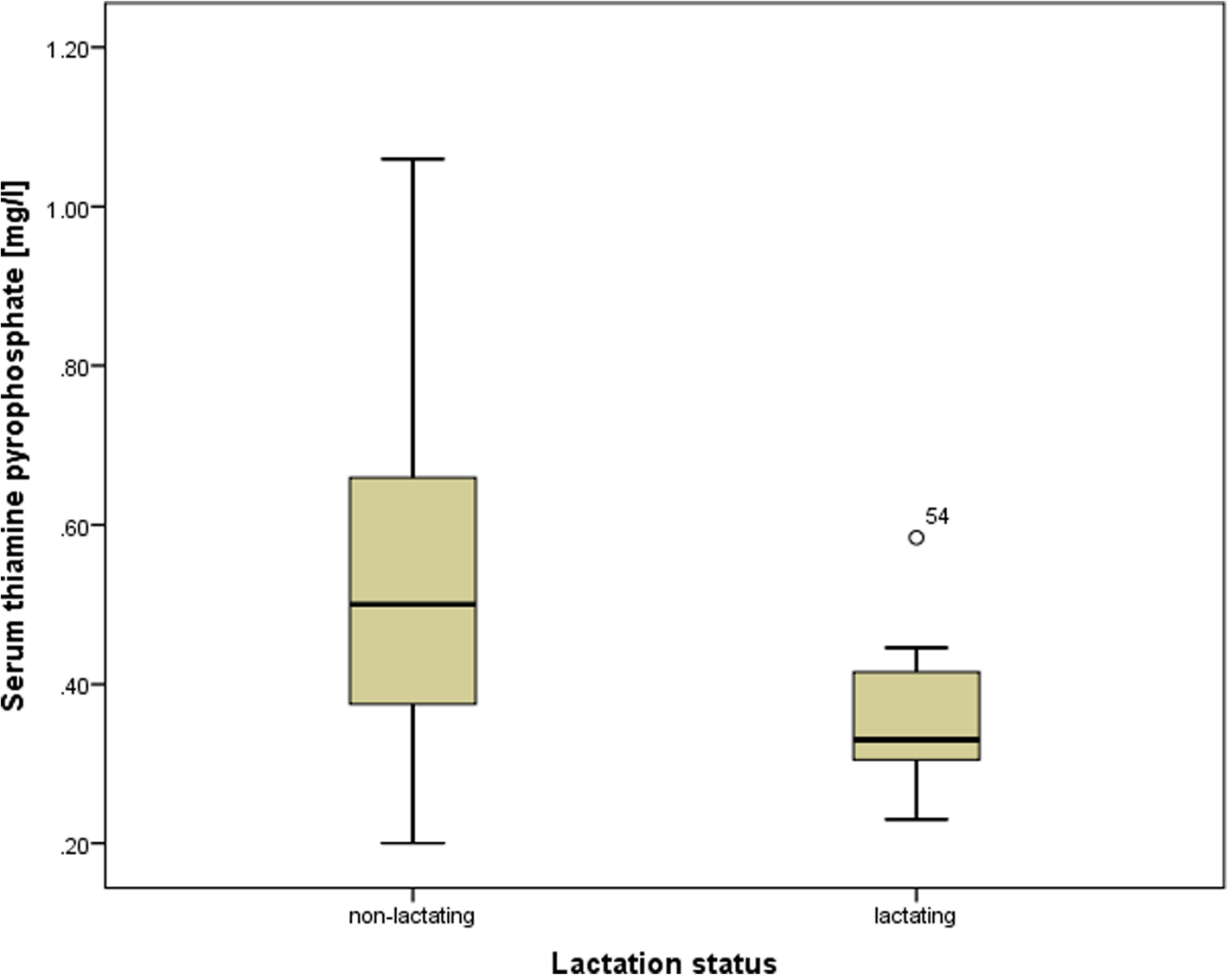
Thiamine pyrophosphate serum concentrations of bottlenose dolphins (*Tursiops truncatus*) in relation to lactation status. *Box* plots comparing thiamine pyrophosphate serum concentrations in lactating and non-lactating bottlenose dolphins (*Tursiops truncatus*). *N* = 7 for lactating females, *N* = 47 for non-lactating dolphins. *Box* plots show the range of the mean 50 % of the data. Within the *boxes*, the *thick line* indicates the arithmetic mean. Fifty-four is the number of one dolphin, indicating an outside value

There was also evidence that thiamine blood concentrations increased when fish was thawed without running water, compared to thawing fish under running water or washing fish with running water after thawing (see Fig. 2). However, no significant result was achieved, when the institution was inserted as a fixed effect. This effect was also not noticeable with regard to the other water soluble vitamin measured, cobalamin. Also, thiamine blood concentrations in lactating females were lower than in the other dolphins as shown in Fig. 3. Here too, no significance resulted from a linear model which included the institution as a fixed effect. Finally, no correlation was found between outdoor or indoor animals and their blood concentration of calcidiol.

## Discussion

### Questionnaire

Over 60 % of the institutions fed five or more fish species. Feeding various different species has several advantages. The animals are used to receive a variety of fish, which can help balancing out nutritional differences. Herring, for instance, is a very fatty fish, whereas capelin is lean. Hence fatty fish was fed to lactating animals and lean fish to dolphins which do not require these high levels of energy. Also, a lot of fish species commonly fed to bottlenose dolphins contain the enzyme thiaminase (herring and smelt, among others [3]), which metabolizes thiamine and thus renders most of it unavailable. Thus, feeding different fish species help to cover the loss of one species’ thiamine with another species that still contains enough of this vitamin. However, feeding many different fish species requires facilities which can order large quantities of fish and store these over a long period of time, making it vulnerable to nutrient loss. The goal is to use the freshest fish possible, while still providing a complete diet. One strategy on how this can be achieved is by varying the fish to match seasonal availability, which is ecoconscious and provides the freshest fish. So far, this strategy has been implemented by only one institution.

Fourteen institutions checked the origin and the capture date of the fish. While food generally can be divided into protein, fats, carbohydrates, and water plus minerals, vitamins and fibre, some of these components (mainly fat and water) vary greatly in fish. Various factors, like age of the fish and season of the year have an influence on the composition [3]. In herring, for example, the fat content differs between 2 and 4 % in spring to 15–20 % in winter per whole fish [3]; in capelin from Newfoundland, it drops from 18 % in December to 3 % in June [8]; and in mackerel caught off the coast of Portugal, it shifts from 1.4 % in February to 7.5 % in August [9]. These extreme changes show that calculating a balanced diet and meeting daily requirements for fish eating animals is difficult and should be constantly adapted. By checking the history of the fish, a lot of information can be acquired. Similarly, checking nutritional values with every new batch is also crucial, as nutritional composition is highly variable; all the participating institutions realized these problems. While most of them tested caloric and fat content, protein and peroxide, vitamin content of whole fish was not tested by any of the institutions, mostly for cost reasons. It is very expensive, but would be a good tool to assess the need of vitamin supplementation, mostly of the fat soluble vitamins.

Moving on to the questionnaire section storing and thawing fish. All facilities ordered fish block frozen. As one block only contains one species of fish, feeding five or more species of fish means 5 or more blocks of frozen fish placed in the storage facility. This potentially leads to longer storage time and thus a deterioration of the fish’s nutritive quality. A study has shown that vitamin A declines significantly over a storage time period of 9 months [10]. Additionally, a long storage time can also be potentially hazardous. In the centre of whole blocks of frozen fish, microbial growth or rancidity might occur due to the freezing mechanism. Blocks get frozen from the outside, creating a temperature gradient to the fish in the centre and leading to a possible problem by not completely freezing the fish.

Optimal temperature of the storage freezer is below −18 °C, and for fish stored for more than 6 months −23 °C [2]. According to another study, freezer temperature between −2 and −9 °C lead to critical nutrient loss and long-time storage at +6 °C results in unacceptable food [2]. It is also crucial to maintain a high humidity in freezer spaces, so that dehydration losses can be kept at a minimum [2]. A high level of humidity is essential, because fish is the main source of water for dolphins. Careful freezing and thawing can minimize water losses and, with that, loss of water soluble nutrients. Since fish is known to lose the least water when thawed in a refrigerator [2] and since thawing at room temperature and under running water leads to a higher microbial build up and to a quicker deterioration of the quality of the fish, it is essential to thaw fish in a refrigerator. Thawing under running water also leads to the flushing out of nutrients.

Six facilities cut their fish in to smaller pieces before feeding. This is generally not necessary, as dolphins need the nutrients of the whole fish. Cutting fish into pieces for feeding leads to nutrient loss through the juice seeping from surfaces and can create a hygienic problem. Once thawed, fish should be used within 24 h to guarantee the freshness of the fish [2].

A point of consideration is the fact that the questionnaire was sent only to EAAM-Members. This could potentially lead to a bias in the study, as there are eight additional institutions in the EU that are not members of the EAAM. 19 out of the 26 EAAM-Members participated in this study. They follow specific guidelines like e.g. food preparation and handling must be conducted so as to ensure the wholesomeness and nutritive value of the food. Frozen fish must be stored in freezers that are maintained at a maximum temperature of −18 °C (0 °F). The length of time food is stored and the method of storage, the thawing of frozen food, and the maintenance of thawed food must be conducted in a manner that will minimize contamination and that will assure that the food retains nutritive value and wholesome quality until the time of feeding” (Current EAAM Standards and Guidelines). Accordingly, it is possible that the questionnaire provides a false positive view by only including members of the EAAM, which are provided with a minimum storage freezer temperature with their guidelines. Yet the storage time and thawing procedure do not follow an exact protocol. The reason only EAAM members were questioned in this study, was that only one subspecies of bottlenose dolphins was used (*Tursiops truncatus truncatus*). Most facilities that are not members of the EAAM keep dolphins from the black sea, *Tursiops truncatus ponticus*, which are believed to be a subspecies of *Tursiops truncatus*.

### Blood values

The results for vitamin A, retinol, suggested that the current intake of vitamin A in the ten tested facilities is high, since the confidence interval for animals in human care was 27 % higher than that of free-ranging animals. This finding suggests that vitamin A stores are saturated. It is first stored in the liver and in fatty tissues, like blubber, and blood concentrations only start to rise when the storage capacity is full. It has been shown that polar bears store 80 % of the total retinol in lipid droplets in the liver [11]. The liver is the main storage for vitamin A in many species, except for those that have other significant fat depots, like marine mammals. A study of seals showed that the concentrations of vitamin A in the blubber of the different subspecies of seals were roughly 10–20 % of the concentrations in the liver [12]. If equalled out for body mass, the blubber is responsible for 50–60 % of the long term storage of vitamin A. Thus, the blubber and the liver play a very important role in the storage of vitamin A.

Since fish is high in vitamin A, additional supplementation is not advisable in such high quantities as multivitamin tablets provide. There are many reports of vitamin A toxicity in humans and other species [13]. However, no reports for marine mammals or other fish eating mammals exist to our knowledge and it is unclear, whether high retinol blood concentrations have a direct negative impact on the animal. A study showed that a high vitamin A supplementation decreases vitamin E uptake in northern fur seals [6]. 50,000 IU of retinol per animal per day had a negative effect on vitamin E uptake. The same has been shown in mice, where a high vitamin A supplementation interfered with the absorption of vitamin E and D3 [14]. Further, a deficiency of vitamin E and D3 is known to have a negative impact on the animal. If daily supplier’s recommendations are followed, one of the multivitamin tablets used in European facilities contains 50,000 IU retinol and may thus may have a negative effect on vitamin E and vitamin D3 uptake. Vitamin A concentrations in liver or blubber of bottlenose dolphins are still unknown and further investigations on the retinol storage capacity of dolphins are necessary. From the present results, it is recommended not to exceed 50,000 IU of vitamin A per animal per day when using multi-vitamin tablets.

For thiamine pyrophosphate (vitamin B1), findings of this study suggests that the thawing technique influences thiamine blood concentration. The fact that a diet of raw fish, without supplementation of thiamine, leads to a deficiency is well established as this has been shown in California sea lions [15] and Atlantic bottlenose dolphins [16]. Thiamine deficiency in fish eaters is mainly attributed to the fact that many different fish species contain the enzyme thiaminase, which metabolizes thiamine during storage [17]. The results of this study show that thiamine might also be lower in fish because of thawing methods that make use of running water. However, the thawing techniques, fish species, fish origin and vitamin supplementation in these ten facilities have not been standardised and cobalamin (vitamin B12), the other water soluble vitamin tested in this study, was not influenced by the thawing method. More testing is required in a standardised setting to prove that the thawing method influences the concentration of water soluble vitamins.

Another finding for thiamine was a lower concentration in lactating females, although no significance was achieved. A study in seals found that pregnant and lactating seals were 2.5 times more likely to develop thiamine deficiency than other female seals [18]. Lactating and maybe pregnant female dolphins should be supplemented with more thiamine than non-breeding dolphins. The latter should receive 25 mg per kg fed fish per day, while lactating female bottlenose dolphins should receive more.

To our knowledge, no reference concentrations for cobalamin (vitamin B12) in bottlenose dolphins exist. B12 is a water-soluble B-vitamin with a key role in the normal functioning of the brain and nervous system as well as for the formation of blood.

Calcidiol (vitamin D3) values, compared to references from free ranging animal, are low in this study. Such levels were unexpected, as fish contains a lot of vitamin D3 and this vitamin is not known to deteriorate during freezing or with long storage time [2]. Multiple factors could explain the low serum vitamin D3. The reference values found for bottlenose dolphins were tested by using a different laboratory analysis and the number of animals tested in these studies was very small [19, 20]. Also, the shown low concentrations could also be due to the fact that excess vitamin D3 is stored in the blubber or excreted, as a study in hooded seals shows [21], or that excess vitamin E and vitamin A have a negative effect on vitamin D3 uptake, as has been shown in mice [14]. Evaluations of whole frozen fish over time and dolphin blubber for vitamin D3 content in addition to supplementation and serum concentrations might help to determine losses, requirements and over-supplementation. No negative health implications from low serum calcidiol concentrations have been detected.

Vitamin E (tocopherol) is 53 % higher in the 57 tested animals, compared to free-ranging animals in Sarasota Bay, Florida [7]. In contrast to vitamin A, where toxicity reports are frequent in species other than marine mammals [13], reports due to excess vitamin E intake in general are rare. One of the existing reports describes a case, where excessive vitamin E supplementation induced a vitamin K responsive coagulopathy in pink-backed pelicans [22]. A study of mice showed, that excess vitamin E inhibits the uptake of vitamin D3 and improved the uptake of vitamin A [14].

Serum vitamin E is approximately proportional to dietary intake over a longer period of time in humans [23] and in ringed seals [12]. In the latter, there was a rapid rise in tocopherol in the liver after administration of radioactive α-tocopherol, followed by a rapid decline within 24 h. In the blubber, only a slight but long-term increase of tocopherol was noted after administration of radioactive α-tocopherol [12]. The blubber appears to be an important long term storage location in seals [24]; the same is probably true for bottlenose dolphins. As the blubber is mostly fat and contains long-chained polyunsaturated fatty acids, vitamin E gets stored in the blubber and acts as an effective antioxidant on site. The findings of this study suggest that the supplementation of tocopherol (vitamin E) may be excessive due to serum concentrations of tocopherol and should be reduced because of the vitamin interactions mentioned above. However, it is shown that there is a need for vitamin E supplementation due to losses from storing and thawing [2] and no clinical signs were observed in the animals. The recommended supplementation of 100 IU of vitamin E per kg fed fish per day should be implemented.

## Conclusions

1. Thawing fish with running water seems to have a negative effect on thiamine pyrophosphate (vitamin B1) blood concentrations. However, further testing is required.
2. Lactating bottlenose dolphins (*Tursiops truncatus*) seem to have a higher need for thiamine; therefore they should receive extra supplementation of vitamin B1.
3. Findings suggest an over-supplementation of retinol (vitamin A) and tocopherol (vitamin E) in bottlenose dolphins because of high concentrations in vitamin supplements. Therefore vitamin A supplementation should not exceed 50,000 IU per animal per day and tocopherol supplementation should be 100 IU of vitamin E per kg fed fish per day.

## Additional file

Additional file 1: A: Questionnaire. Questionnaire sent to 25 institutions that keep bottlenose dolphins (*Tursiops truncatus*) to assess fish-handling techniques and vitamin supplementation. (PDF 88 kb)

## Abbreviations

ANOVA: Analysis of variance
CLIA: Chemiluminescence immunoassay
EAAM: European association of aquatic mammals
ECLIA: Electro- chemiluminescence immunoassay
EDTA: Ethylenediaminetetraacetic acid
HPLC: High-performance liquid chromatography
U-HPLC: Ultra high-performance liquid chromatography
