# Supplementary material for: Vitamin blood concentration and vitamin supplementation in bottlenose dolphins (Tursiops truncatus) in European facilities
Source: BMC Vet Res. 2016 Sep 5;12(1):180. doi: 10.1186/s12917-016-0818-1 (PMC5011994; doi:10.1186/s12917-016-0818-1)
Supplement: Additional file 1: — A: Questionnaire. Questionnaire sent to 25 institutions that keep bottlenose dolphins (Tursiops truncatus) to assess fish-handling techniques and vitamin supplementation. (PDF 88 kb) [file 12917_2016_818_MOESM1_ESM.pdf]

Questionnaire in order to study the vitamin status of bottlenose dolphins (Tursiops truncatus) in European facilities

For my doctoral thesis I am conducting a survey to clarify the vitamin status of bottlenose dolphins in European facilities in collaboration with the Institute of Animal Nutrition in Zurich, Switzerland, the Tiergarten Nürnberg, Germany, and the Zoo Duisburg, Germany. The following questionnaire was designed to obtain the data necessary for this research. The project will contribute to the field of zoological medicine by providing information on blood levels of healthy animals as well as insights to effective supplementation. It will also be providing data to asses the oral vitamin supplementation in captive-held bottlenose dolphins. If you choose to add your email-address at the end of the questionnaire, I will be happy to send you the results. In addition, the goal of this questionnaire is to find suitable candidates for vitamin-blood-level testing. If your facility intends on collaborating in this field as well, please do not hesitate to contact me directly. You will then know the direct effect of your vitamin supplementation. Thank you very much in advance for your collaboration and your help. It would be greatly appreciated if you could fill out and return the questionnaire before March 2012.

Yours sincerely,

Angela Gimmel, doctoral student, University of Zurich

Date:_________________

This questionnaire is divided in 9 different sections, starting from ordering and purchasing fish, to storing, thawing and feeding. If you turn the whole form over, there is a small section about vitamin supplementation. It would be greatly appreciated if the staff veterinarian could fill this out first. The other parts of the questionnaire are mainly designed for the dolphin staff, although a collaboration of the staff veterinarian and the dolphin staff with regard to the entire form is greatly encouraged. All in all, there are 49 questions and it will take you approximately 20min to fill it out completely.

1. Ordering and purchasing fish:

This first section in the fishes' journey from buying to feeding is about ordering and purchasing fish. We would like to gather information about the fish you are purchasing for your dolphins.

1. What species of fish are you feeding your bottlenose dolphins? Please tick all that apply:

- ☐ Herring
- ☐ Mackerel
- ☐ Smelt
- ☐ Squid
- ☐ Sprat
- ☐ Cod
- ☐ Whitling
- ☐ Angler
- ☐ Other:____________________________

2. Does the fish vary seasonally?

- ☐ Yes
- ☐ No (please continue with question 5)

3. If yes, please note the fish you are feeding according to each season:

- ☐ Spring (March - May):____________________
- ☐ Summer (June - August):____________________
- ☐ Autumn (September - November):____________________
- ☐ Winter (December - February):____________________

4. Do you control the history of the fish that was caught for your bottlenose dolphins?

Please tick yes or no for every line:

We control...	yes	no
… where the fish was caught		
… when the fish was caught		
… current contamination problems		
… other:		

5. Where do you purchase the fish for your dolphins? Please tick all suppliers that apply or write down your own ones.

- ☐ Hassel-Lang
- ☐ Farell
- ☐ __________
- ☐ Parleviet & van der Plas
- ☐ __________
- ☐ __________

6. How is the fish frozen that you purchase for your bottlenose dolphins?

- ☐ Block frozen (meaning an amount of fish frozen in one block)
- ☐ Individually quick frozen
- ☐ Other: ____________________

7. What is the weight of the fish-packs you mostly purchase?
   - ☐ 1-5kg per pack, approximately ____kg
   - ☐ > 5-10kg per pack, approximately ____kg
   - ☐ > 10-20 kg per pack, approximately ____kg
   - ☐ > 20-40kg per pack, approximately ____kg
   - ☐ More than 40kg per pack, approximately ____kg
8. How long does a single pack last in average?
   - ☐ 1 day
   - ☐ 2 days
   - ☐ 3-4 days
   - ☐ More than 4 days
9. Does the package size vary with the size of the fish?
   - ☐ Yes, we use small packages for small fishes and large packages for big fishes
   - ☐ Yes, we use large packages for small fishes and small packages for big fishes
   - ☐ No

2. Inspection of shipment

This section is about the inspection of the shipment, i.e. when the truck is bringing your fish delivery, what do you do before transporting the fish to your storage area.

1. Do you inspect the fish shipment before storing it? Please tick yes and no for every line.

We check...	yes	no
… the documentation		
… if there are non food items on the truck		
… that the temperature was maintained during shipment		
… for optical signs of thawing and refreezing		
… the fish manually for firmness and smell		
… other:________________________________		

2. Do you conduct nutritional analyses before feeding the fish to the bottlenose dolphins?
   - ☐ Yes
   - ☐ No (continue with point 3. Storage)
3. If yes, how often do you conduct them?
   - ☐ Once a year
   - ☐ Twice a year
   - ☐ Every three months
   - ☐ Other: ________________________________

4. Please note the data you obtain from your analyses (tick all that apply)

☐ Fat content ☐ Protein ☐ Crude ashes

☐ Vitamin content ☐ Mineral content ☐ Other:_______________

3. Storage

In this section we are interested in how you are storing your fish for your bottlenose dolphins.

1. What is the temperature (in ° Celsius) of your storage freezer? ______________________
2. Do you store other items than fish in your storage freezer area?

   ☐ Yes

   ☐ No (continue with question 4)
3. If yes, please note the items

   ____

   ____
4. What is the maximum time frame fish is stored in your storage freezer?

   ☐ 1 - 2 months ☐ 7 - 9 months ☐ More than 12 months

   ☐ 3 - 6 months ☐ 10 - 12 months ☐ Other:_____________
5. Does the storage time depend on the species of fish you are storing?

   ☐ Yes, we store ____the longest

   ☐ No

4. Transport

In this short chapter we would like to know about the transport of the fish from the freezer to the thawing/kitchen/feeding area.

1. Are the storage freezer and the thawing/kitchen/feeding area in the same building?

   ☐ Yes (please continue with section 5 Thawing)

   ☐ No
2. Do you monitor the temperature during the transport from the storage freezer to the thawing/kitchen/feeding area?

   ☐ Yes

   ☐ No (please continue with section 5 Thawing)
3. If yes, please note the measures you take to keep the fish frozen ____________________

   ____

   ____

5. Thawing

This chapter focuses on thawing, a process we are very interested in.

1. What method do you use to thaw your fish? Please tick all that apply.
   - ☐ In a refrigerator
   - ☐ Under running water (please note the temperature of the running water: ___________)
   - ☐ In a microwave oven (please note the temperature of the microwave oven: _________)
   - ☐ At room temperature (please note the timespan the fish usually needs to thaw: _____)
   - ☐ Other: ____
2. If you use a refrigerator, what is the temperature range?
   - ☐ 0-1°C
   - ☐ 2-4°C
   - ☐ 5-8°C
   - ☐ Other: ____
3. How soon do you feed the fish after it is removed from the storage freezer?
   - ☐ Within 12 hours
   - ☐ Within 24 hours
   - ☐ Within 48 hours
   - ☐ Other: ____
4. Do you cut large packages of fish into pieces before thawing?
   - ☐ Yes
   - ☐ No

6. Handling thawed fish

In this part the focus lies on cutting the fish, as water soluble vitamins are lost in the cutting juice seeping from the fish.

1. Do you keep the fish cool (at 4°C) between thawing and processing/feeding?
   - ☐ Yes
   - ☐ No
2. Do you cut the fish before feeding?
   - ☐ Yes
   - ☐ No (please continue with section 7 Feeding)
3. If yes, are you cutting anything away before feeding? I.e. not feeding to your dolphins
   - ☐ Yes, we are cutting away: ____
   - ☐ No

4. Why are you not feeding the parts you mentioned above? ___________________________
____
____

5. If you handle the fish in any other way not mentioned above, please note below. _______
____
____

7. Feeding

Last stop in the fishes' journey from purchasing to feeding is the feeding of the fish to your animals.

1. How often do you feed your bottlenose dolphins?
   - ☐ Once a day
   - ☐ Twice a day
   - ☐ Other:____

2. Do you feed your dolphins individually?
   - ☐ Yes
   - ☐ No

3. Do you vary the diet for each animal when it is...

	Yes, we change...	no
… sick/injured?		
… old?		
… a juvenile?		
… pregnant?		

4. Who is responsible for setting the formulation of the diets for the dolphins? Please tick all that apply

☐ Veterinarian ☐ Nutritionist
☐ Chief trainer ☐ Other:________________________________

5. Are you supplementing vitamins?
   - ☐ Yes
   - ☐ No (please continue with section 8 General Information)

6. If yes, please note the kind of supplements you use. Please tick all that apply or write down your own ones if there not on the list

☐ SeaTabs ☐ Individual Vitamins ☐ _______________
☐ Mazuri Fish Eater Tablets ☐ __________________ ☐ _______________

7. Please note one daily vitamin supplementation (average from the last 2 weeks) for each dolphin or attach it as separate sheets to the questionnaire:

Name of the dolphin	Supplementation	Dosage (in amount of tablets and if known in mg)

8. Are you supplementing anything else? (please note the brand name)

☐ Yes, namely ____
____

☐ No

8. General information:

Finally it would be greatly appreciated if you could fill out some general information about you, your facilities and your dolphins. The data will be handled strictly confidentially.

1. Would you like to be informed about the results of this project?

   ☐ Yes ☐ No
2. Your name and position at the facility: ______________________________
3. Name, contact address of the facility and, if you want to be contacted about the results, please note your email-address: ______________________________
   ____
   ____
4. Number of bottlenose dolphins kept at your facility: ____________________
5. Please note the name, sex and date of birth of each dolphin that lives in your facility at the moment:

Name	Sex	Date of birth (if known)

6. Please note the size of the enclosure (length[m] x width[m] x depth[m]) and if known the amount of water it holds. Please specify if it is indoor or outdoor. If there are more than one, please note the size of each of the enclosures, the number of animals in each of them as well as if it is indoor or outdoor: ______________________________________
____
____

9. Health management (Section to fill out by the veterinarian)

1. Is there any recommendation, publication, documentation you rely on how to supplement your dolphins with vitamins? Please tick all that apply or write down the ones you use

☐ CRC Handbook of Marine Mammal Medicine	☐ ______________	☐ ________________
	☐ ______________	☐ ________________
☐ ____________________	☐ ______________	☐ ________________

2. Have you taken blood once to ascertain the vitamin status of your animals?
   - ☐ Yes
   - ☐ No (Please continue with question 4)
3. If yes, please tick all the values you have checked

☐ Vitamin A	☐ Vitamin B12	☐ Vitamin D3 (1.25-di-OH)
☐ Vitamin B1	☐ Biotin	☐ Vitamin D3 (25-OH)
☐ Vitamin B2	☐ Folin	☐ Vitamin E
☐ Vitamin B6	☐ Vitamin C	

4. How often do you take blood samples from your animals as a matter of routine?
   - ☐ Once a year
   - ☐ Every half year
   - ☐ Every three months
   - ☐ Other: ____
5. Where do you send your blood samples for testing? ______________________________
____

Thank you very much for filling out the form. Please return it via mail or email to:

Angela Gimmel
Iddastrasse 49
9008 St. Gallen
Switzerland
emilia_ricarda@gmx.net
